# Irregular delay of adjuvant chemotherapy correlated with poor outcome in stage II-III colorectal cancer

**DOI:** 10.1186/s12885-022-09767-y

**Published:** 2022-06-18

**Authors:** Yuanyuan Chen, Mingyue Xu, Qianwen Ye, Jia Xiang, Tianhui Xue, Tao Yang, Long Liu, Bing Yan

**Affiliations:** 1 Department of General Medicine, Hainan Hospital of Chinese PLA General Hospital, Sanya City, Hainan P.R. China; 2 Department of General Surgery, Hainan Hospital of Chinese PLA General Hospital, Sanya City, Hainan P.R. China; 3 Department of Oncology, Hainan Hospital of Chinese PLA General Hospital, No. 80 of Jianglin Road, Haitang District, Sanya City, Hainan province 572000 P.R. China; 4 Department Traditional Chinese Medicine, Tianyou Hospital of Tongji University, No. 528 of Zhennan Road, Putuo District Shanghai, 200331 P.R. China

**Keywords:** Colorectal cancer, Adjuvant chemotherapy, Delay, Disease-free survival

## Abstract

**Aims:**

Adjuvant chemotherapy (ACT) plays an important role in improving the survival of stage II-III colorectal cancer (CRC) patients after curative surgery. However, the prognostic role of irregular delay of ACT (IDacT) for these patients has been less studied.

**Materials and methods:**

A total of 117 stage II-III CRC patients who underwent radical resection and received at least 3 months ACT were enrolled retrospectively. The significance of IDacT, including total delay (TD) and delay *per* cycle (DpC), in predicting disease-free survival (DFS) was determined using receiver operating characteristic curve (ROC) analysis. The survival differences between the TD, DpC-short and DpC-long subgroups were tested using Kaplan–Meier analysis, and risk factors for prognosis were determined using a Cox proportional hazards model.

**Results:**

Using 35.50 and 3.27 days as the optimal cut-off points for TD and DpC, respectively, ROC analysis revealed that TD and DpC had sensitivities of 43.60% and 59.00% and specificities of 83.30% and 62.80%, respectively, in predicting DFS (both P < 0.05). No differences in the clinicopathological parameters were found between the TD, DpC-short or -long subgroups except histological differentiation in different TD subgroups and combined T stages in different DpC subgroups (both *P* = 0.04). Patients in the TD or DpC-long group exhibited significantly worse survival than in the -short group (TD: Log rank = 9.11, P < 0.01; DpC: Log rank = 6.09, *P* = 0.01). DpC was an independent risk factor for prognosis (HR = 2.54, 95% CI: 1.32–4.88, *P* = 0.01).

**Conclusions:**

IDacT had a profound effect on the outcome for stage II-III CRC. Although TD and DpC were significant for the prognosis, DpC was more robust, and patients who presented DpC for a long time had a significantly worse DFS.

## Introduction

Adjuvant chemotherapy (ACT) improves survival in stage II-III colorectal cancer (CRC) patients, and the efficacy of chemotherapy regimens based on oxaliplatin, such as XELOX (oxalipaltin + capecitabine) and FOLFOX (oxalipaltin + leucovorin + 5-fluorouracil), were extensively validated in a series of clinical trials [1–3]. Six months (m) of ACT was the standard of treatment for these patients, but a shortened duration of ACT of 3 m was acceptable for both disease-free survival (DFS) and overall survival (OS) for certain cases due to the significant adverse effects (AEs) of chemotherapy, as validated in recent studies [2–7]. However, many factors could impair the benefits of ACT in practice.

Delayed ACT (DacT) conventionally refers to the interval between the points of surgery and the initiation of ACT, which is common in the clinic. DacT has been documented in many malignancies, including gastric cancer [8, 9], breast cancer [10, 11], lung cancer [12] and CRC [13–15]. Notably, DacT consistently correlated with poor outcome in these studies [8–15]. Kim et al. investigated the impact of DacT (defined as ≥ 8 weeks (w)) on survival in stage II-III CRC and found that DacT was associated with inferior OS [13]. Becerra et al. used a similar cut-off point to examine the impact of DacT in stage III cases on DFS, and the results indicated that DacT was correlated with poor DFS [14]. The underlying causes for DacT are complicated and include patient factors (e.g., sex, age, comorbidity, postoperative anxiety, and depression), insurance status, socioeconomic status, and treatment-related factors (e.g., emergency surgery, reoperation, and prolonged hospital stay) [11, 13, 16–18]. However, DacT also occurred during the prescribed ACT with high probability due to additional cause-AEs of chemotherapeutic drugs [16]. Although the specific rate of DacT during treatment is largely unknown, those of grade 3 or 4 AEs were 37.6% and 24.2% in the 3-m arms of the FOLFOX and XELOX regimens, respectively, and increased to 56.9% and 36.9% in the 6-m arms according to IDEA research [19]. These AEs resulted in a high probability of dose adjustment and DacT in practice. Notably, the DacT during these treatments may be highly irregular due to the different recovery capabilities of the patients. However, whether this irregular DacT (IDacT) affected the survival of the patients was not addressed.

The present study examined the effect of IDacT, including total delay (TD) and delay *per* cycle (DpC), on the outcome in stage II-III CRC.

## Materials and methods

### Patient enrollment

CRC patients treated in Hainan Hospital of Chinese PLA General Hospital were retrospectively enrolled from December 2012 to December 2021. Patients were included if they met the following criteria: 1. curative resection of the primary lesion; 2. pathologically confirmed as stage II-III (according to the 8^th^ edition of American Joint Committee on Cancer) with no suspicious distant metastasis; and 3. a clear ACT record (at least 4 cycles ACT based on XELOX or single capecitabine (X), or at least 6 cycles ACT based on FOLFOX). Patients who met any of the following criteria were excluded: 1. receiving any preoperative neoadjuvant therapies; and 2. loss or refusal to follow-up or a follow-up period less than 36 m. The risk factors, including lymphovascular invasion, histopathological poorly differentiated, T4 or obstruction [20], and other clinicopathological information, were collected as previously described [21, 22]. The study was conducted in accordance with the principles stated in the Declaration of Helsinki and was approved by the ethics committee of Hainan Hospital of Chinese PLA General Hospital (ID: 301HLFYLS15), and informed consent was obtained from the patients or their authorized relatives.

### Definition of TD, DpC and DFS

**Fig. 1:**
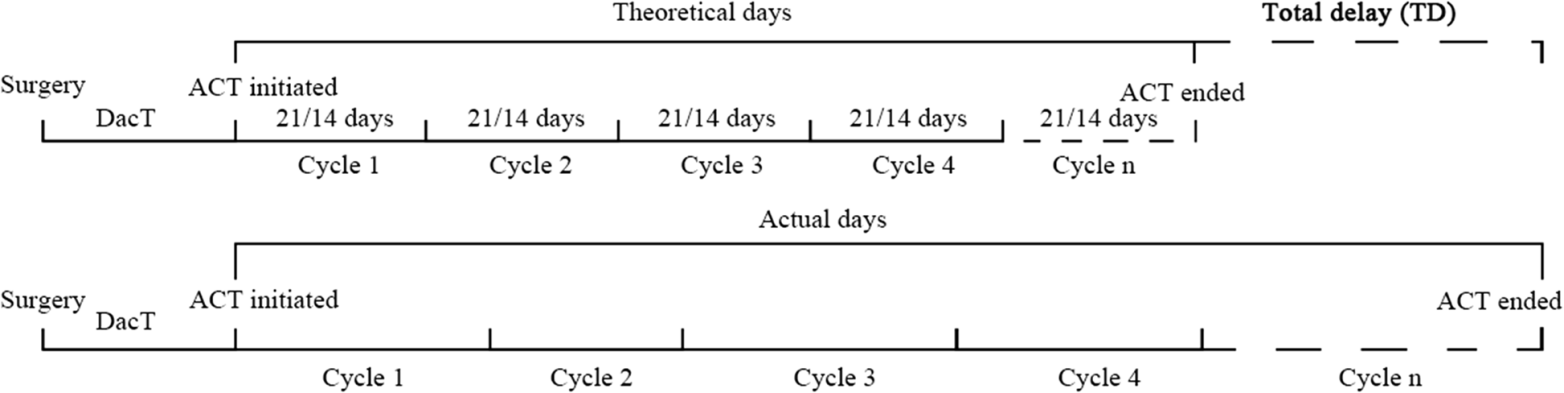
Methods to estimate the total delay

The TD was calculated as the sum of the actual days minus the theoretical days from the initiation of ACT to the first day of the last cycle. The theoretical number of days for which patients underwent regimens such as XELOX or single X was counted every 21 days (d), and it was counted every 14 d for patients receiving FOLFOX (Fig. 1). The days from the day of surgery to the initiation of ACT were not included in the TD calculation. After calculating the TD, the DpC was determined as the TD divided by the actual ACT cycles. DFS was defined from the time of surgery to the date of any recurrence or metastasis or the date of death from any cause. The follow-up was performed as described in a previous report [22], and the latest follow-up point was December 2021.

### Statistical analysis

Statistical analyses were performed using SPSS 20.0 (SPSS Inc., Chicago, IL, USA). The optimal cut-off points of TD and DpC were calculated using receiver operating characteristic (ROC) curve analysis for DFS. Patients were divided into TD, DpC-short or -long subgroups according to the *Youden* index. The differences in the collected clinicopathological parameters between these subgroups were estimated using the χ2-test. Survival differences between these subgroups were assessed using Kaplan–Meier (K-M) analysis followed by the log rank tests. Risk factors for the outcome were calculated using a Cox proportional hazards model. A double-sided *P* < 0.05 was considered statistically significant.

## Results

### Demographic features of the cohort

**Fig. 2:**
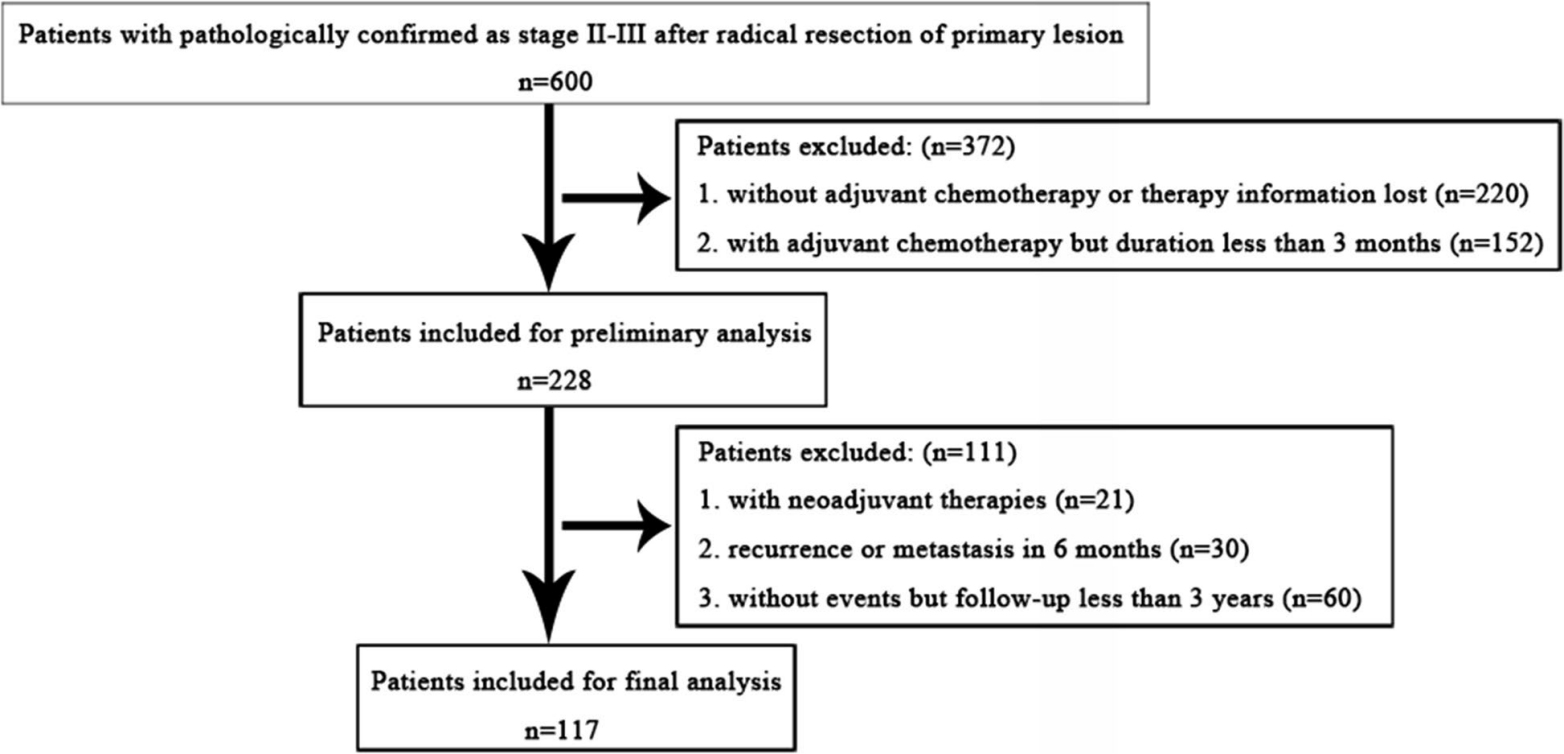
Flowchart of case inclusion and exclusion

Following the inclusion and exclusion criteria, 117 patients were enrolled for the final analysis (Fig. 2). The basic demographic characteristics are shown in Table 1. Briefly, there were 30 stage II (with 16 cases accompanied at least 1 risk factor) and 87 stage III (with 43 cases accompanied at least 1 risk factor) patients. The mean TD and DpC were 24.18 d (range: 0–117 d) and 3.74 days (range: 0–16.60 d), respectively. Five patients presented a conventional DacT (defined as ≥ 8 w) of 61 d (2 cases), 65 d, 66 d and 70 d (1 case each), and the rest of the cases did not exhibit a delay (mean time from surgery to initiation of ACT: 32.60 d, range: 7–79 d). The mean age of the patients was 53.44 years (y) (range: 24–80 y), and the mean follow-up time was 41.80 m (range: 3–60 m). At the end of the follow-up, 39 events occurred, and the 3-year DFS rate was 66.67% (90.00% for stage II and 58.60% for stage III).

**Table 1:** Basic demographic characteristic of the study

Parameters	Cases (n/%)
**Age (y)**	
< 60	81 (69.23)
≥ 60	36 (30.67)
**Sex**	
Male	77 (65.81)
Female	40 (34.19)
**Tumor location**	
Right	33 (28.21)
Left	84 (71.79)
**Tumor morphology**	
Ulcerated type	57 (48.72)
Protruded type	21 (17.95)
Mixed type	6 (5.13)
Unknown	33 (28.21)
**Histological differentiation**	
Well + moderate	92 (78.63)
Poor	25 (21.37)
**Mucinous element**	
With	18 (15.39)
Without	99 (84.61)
**Combined T stages**	
T_1_ + T_2_ + T_3_	94 (80.34)
T_4_	23 (19.66)
**Combined N stages**	
N_0_	30 (25.64)
N_1+_N_2_	87 (74.32)
**TNM stages**	
II	30 (25.64)
III	87 (74.32)
**Tumor deposits**	
With	20 (17.09)
Without	97 (82.91)
**RAS mutation**	
Mutated	6 (5.13)
Wild-type	11 (9.40)
Unknown	100 (85.47)
**MSS status**	
MSI-H	3 (2.56)
MSI-L + MSS	66 (56.41)
Unknown	48 (41.03)
**Ki-67 percentage**	
≥ 75%	41 (35.04)
50–75%	14 (11.97)
0–50%	7 (5.98)
Unknown	55 (47.01)
**Risk factors**	
None	58 (49.57)
1	34 (29.06)
2	15 (12.82)
3	9 (7.69)
4	1 (0.86)
**Adjuvant therapy regimens**	
XELOX	71 (60.83)
FOLFOX	11 (9.40)
XELOX + X	17 (14.53)
FOLFOX + X	1 (0.85)
XELOX + FOLFOX	8 (6.84)
X	1 (0.85)
Others	8 (6.70)

### Predicting the efficacy of TD and DpC for survival and differences in clinicopathological parameters between subgroups

**Fig. 3:**
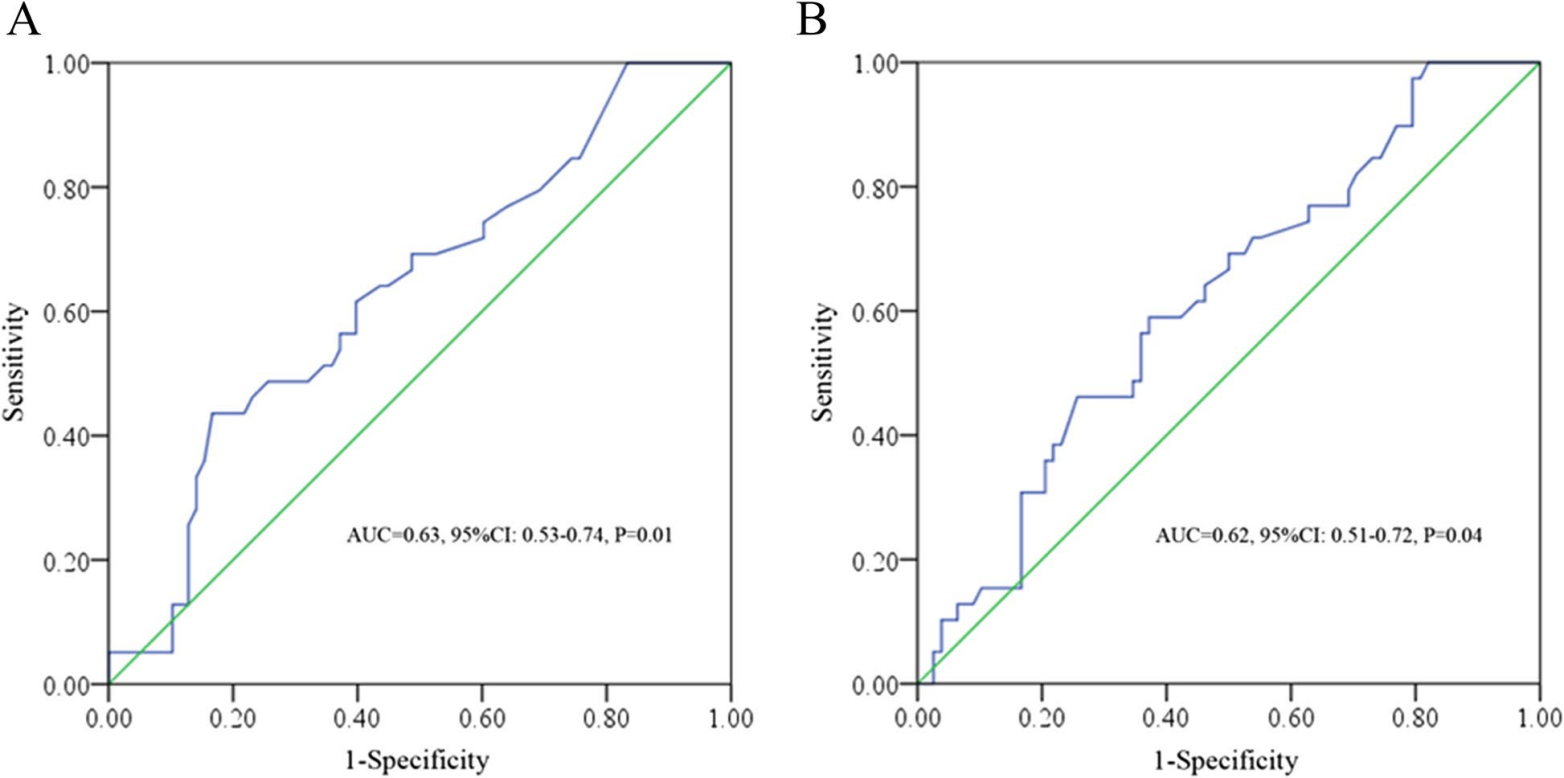
ROC analysis of TD (**A**) and DpC (**B**) in predicting DFS

The *Youden* index from the ROC tests described 35.50 and 3.27 d as the optimal cut-off points for TD and DpC, respectively. The TD and DpC had a sensitivity of 43.60% and 59.00% and a specificity of 83.30% and 62.80% in predicting DFS (the areas under the curve (AUC) = 0.63, *P* = 0.01; AUC = 0.62, *P* = 0.04, respectively) (Fig. 3). Patients were divided into TD-short (< 35.50 d) or TD-long (≥ 35.50 d), DpC-short (< 3.27 d) or DpC-long (≥ 3.27 d) subgroups. Notably, no significant differences were found for the collected clinicopathological parameters between these subgroups except histological differentiation in different TD subgroups and combined T stages in different DpC subgroups (Table 2).

**Table 2:** Differences of clinicopathological parameters among TD, DpC-short and -long subgroups

	**TD**			**DpC**		
	Short	Long	P	Short	Long	P
**Age (y)**			0.37			0.84
< 60	58	23		45	36	
≥ 60	29	7		19	17	
**Sex**			0.27			0.17
Male	60	17		46	31	
Female	27	13		18	22	
**Tumor location**			1.00			1.00
Right	25	8		18	15	
Left	62	22		46	38	
**Tumor morphology**			0.26			0.23
Ulcerated type	44	13		36	21	
Protruded type	12	9		9	12	
Mixed type	5	1		4	2	
Unknown	26	7		15	18	
**Histological differentiation**			0.04^*^			0.37
Well + moderate	64	28		48	44	
Poor	23	2		16	9	
**Mucinous element**			0.08			0.80
With	10	8		9	9	
Without	77	22		55	44	
**Combined T stages**			0.11			0.04^*^
T_1_ + T_2_ + T_3_	73	21		56	38	
T_4_	14	9		8	15	
**Combined N stages**			0.81			0.84
N_0_	23	7		17	13	
N_1+_N_2_	64	23		47	40	
**TNM stages**			0.81			0.84
II	23	7		17	13	
III	64	23		47	40	
**Tumor deposits**			0.78			0.63
With	14	6		12	8	
Without	73	24		52	45	
**Risk factors**			0.14			1.00
None	40	19		32	27	
≥ 1	47	11		32	26	

^*^with significant difference

### Survival differences between TD and DpC subgroups

**Fig. 4:**
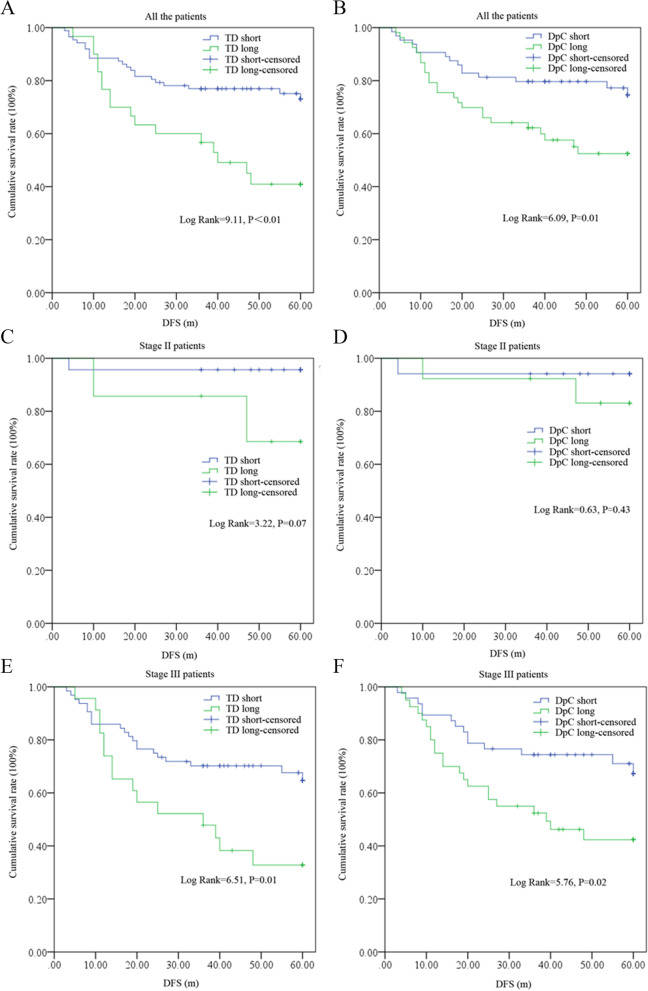
Survival differences between TD, DpC-short or -long subgroups. **A** TD in all the patients; **B** DpC in all the patients; **C** TD in stage II patients; **D** DpC in stage II patients; **E**TD in stage III patients; **F** DpC in stage III patients

K-M analysis showed significant differences in survival between the TD, DpC-short and -long subgroups, and patients in the TD (Log rank = 9.11, P < 0.01) or DpC (Log rank = 6.09, *P* = 0.01)-long groups exhibited significantly inferior survival compared with that of the -short groups. Further, when stage II and III cases were analyzed individually, stage III patients presented a consistent statistical differences in survival among TD, DpC-short and -long subgroups; however, no such differences were found in stage II cases but with a trend of difference only in TD-short and -long subgroups (Fig. 4).

### Univariate and multivariate tests of risk factors for prognosis

Univariate tests found that the combined N stage, TNM stage, tumor deposits, TD and DpC were risk factors for prognosis, and when these parameters were included in multivariate tests, DpC was an independent risk factor for prognosis (HR = 2.54, 95% CI: 1.332–4.88, *P* = 0.01) (Table 3).

**Table 3:** Univariate and multivariate analyses of different parameters for DFS

	**Univariate**			**Multivariate**		
	**P**	**HR**	**95%CI**	**P**	**HR**	**95%CI**
**Age (years)**						
< 60	1					
≥ 60	0.19	1.54	0.81–2.95			
**Sex**						
Male	1					
Female	0.08	0.57	0.30–1.07			
**Tumor location**						
Right	1					
Left	0.50	0.78	0.37–1.63			
**Tumor morphology**						
Ulcerated type	1					
Protruded type	0.34	1.47	0.67–3.28			
Mixed type	0.38	1.73	0.51–5.89			
Unknown	0.76	0.88	0.40–1.96			
**Histological differentiation**						
Well + moderate	1					
Poor	0.07	2.58	0.92–7.26			
**Mucinous element**						
With	1					
Without	0.24	0.63	0.29–1.37			
**T stages**						
T_1_ + T_2_ + T_3_	1					
T_4_	0.72	1.16	0.51–2.63			
**N stages**						
N_0_	1			1		
N_1+_ N_2_	0.01^*^	5.07	1.56–16.51	0.01^*^	4.84	1.48–15.81
**TNM stages**						
II	1					
III	0.01^*^	5.08	1.56–16.51			
**Tumor deposits**						
With	1			1		
Without	0.01^*^	0.27	0.14–0.52	< 0.01^*^	0.26	0.13–0.51
**Risk factors**						
None	1					
≥ 1	0.54	1.22	0.65–2.30			
**TD**						
TD-short	1					
TD-long	< 0.01^*^	2.56	1.36–4.83			
**DpC**						
DpC-short	1			1		
DpC-long	0.02^*^	2.20	1.15–4.20	0.01^*^	2.54	1.32–4.88

^*^with significant difference

*TD* total delay, *DpC* delay *per* cycle

## Discussion

The present study demonstrated an obvious IDacT during the treatment of stage II-III CRC patients, and TD and DpC were useful in predicting DFS with relatively high specificities. Patients who presented a long TD or DpC had worse survival than those who presented a short TD or DpC, in particular, in stage III cases. Additionally, the DpC was likely to be a more robust indicator when compared to TD and it was an independent risk factor. To the best of our knowledge, this study is the first report to address the prognostic role of IDacT in CRC.

DacT impairs the outcome in cancer patients, but most studies focused on the period from surgery to the initiation of ACT. Lu et al. performed a systematic review and meta-analysis of 6107 gastric cancer patients after curative resection and found that the initiation of ACT *per* 4-w delay correlated with significant decreases in DFS and OS [8]. Chen et al. investigated 1520 stage II-III gastric cancer patients and found that patients with DacT (defined as commencement of ACT more than 60 d after surgery) also had significantly worse DFS and OS [9]; additionally, patients in the DacT group were less likely to benefit from ACT [9]. Although the definition of DacT was not consistent in CRC, its role in prognosis was not surprising. For example, Cheung et al. enrolled 5617 stage II-III CRC patients and indicated that DacT (defined as ≥ 3 m) predicted poor OS [23]. Bayraktar et al. performed a study of 186 stage II-III CRC patients and suggested that DacT (defined as ≥ 60 d) was associated with worse OS [24]. Most subsequent studies used 8 w as the cut-off point for DacT and found that it correlated with an inferior DFS or OS in stage II-III cases [13, 14, 25]. The underlying causes of DacT have been extensively studied and include a series of aforementioned factors [11, 13, 16–18]. However, taking into consideration the high probability of grade 3 or 4 AEs during chemotherapy [19], a substantial proportion of patients would experience IDacT in addition to these causes, as demonstrated in DacT. Although no previous studies indicated that IDacT would also impair the outcome of the patients, the consolidated results of DacT in prognosis support such a role of IDacT to some extent because they shared similar causes in practice. When using 8 w as the cut-off point, patients with DacT who completed the prescribed ACT were subsequently regarded as TD long to some extent in our study (although no delay occurred during ACT). When these patients (*n* = 5) were included in the analysis following this assumption, the AUC of TD in predicting DFS was even larger (AUC = 0.64, *P* = 0.02, data not shown) in our study.

The underlying mechanisms by which DacT could impair the outcome are largely unknown and commonly attributed to its decreased killing efficacy for micrometastasis. However, the mechanisms of DacT and IDacT may not be identical because chemotherapeutic drugs have an additional profound effect on cancer cells and patients. From this point of review, we speculated that IDacT would impair the outcome for the following reasons. First, it was found that as many as 19.40% of breast cancer patients who underwent ACT could develop hyperglycemia and exhibited a poor DFS [26]; accordingly, the serum insulin level was observed to experience an elevation [27]. In CRC, it was found that 5-fluorouracil (5-Fu) chemotherapy resulted in an elevation of fasting glucose levels [28], which could correspondingly lead to an increase in insulin secretion. Notably, insulin plays multiple roles in the development of CRC. For example, studies have revealed that it can lead to oxaliplatin resistance via activation of the PI3K/Akt pathway in HT-29 cells [29, 30] and contribute to cancer cell progression via the upregulation of ACAT1 [31]; additionally, it can increase proliferation and migration of HCT-116 cells [32]. Based on these results, patients who presented with IDacT were likely to have a high level of serum insulin that could promote disease development, and the residual cancer cells may become refractory to chemotherapeutic drugs due to the delayed administration of the next cycle of treatment. Second, patients with IDacT had a high probability of dose adjustment due to AEs [19]. Although one study indicated that FOLFOX regimens with different doses of 5-Fu as ACT in CRC showed similar efficiency [33], a dose intensity reduction in these regimens could potentially correlate with an attenuated killing effect for the cancer cells and was associated with impaired survival [34]. Third, patients with IDacT were found to exhibit a slow recovery due to various chemotherapy-related hematological toxicities (CRHT). Although no relationship between chemotherapy-associated neutrophil counts and CRC recurrence was found [35], chemotherapy-induced lymphopenia was a validated risk factor for febrile neutropenia and could lead to shorter DFS (< 0.66 × 10^9^/L) and OS (< 0.91 × 10^9^/L) [35] or early death in CRC [36]. In addition, chemotherapy-induced anemia (CIA) was also not uncommon in practice. It was found that anemia (which contributes to tumor hypoxia) can not only lead to chemotherapy resistance but also increase the invasiveness and metastatic potential and decrease the apoptosis of cancer cells [37]. A study indicated that CIA (< 90 g/L) was an independent prognostic factor for both DFS and OS in CRC patients receiving ACT [38]. Based on these reports, it was plausible that patients with IDacT caused by the above reasons would have a poor outcome.

Noticeably, although XELOX and FOLFOX exhibited similar efficacy in ACT [19, 39], the rate of severe AEs and the patients’ cost were obviously higher in FOLFOX than XELOX [19, 39–41]. Moreover, as the XELOX was redelivered every 21 d, and the FOLFOX regimen was redelivered every 14 d [19], we speculated that the XELOX regimen would be the preferred regimen for ACT based on its relatively mild AEs, cost and the lower frequency for chemotherapy booking. In addition, as an increased IDacT resulted in poor survival, some approaches could be used to minimize its impact on survival or decrease its incidence based on our aforementioned reasons. For example, metformin (a classical agent for the treatment of diabetes mellitus) could be used to control the fluctuation of glucose and it was also found to be effective in reversing insulin-induced oxaliplatin resistance in human colon cancer HCT-116 cells [42]; and clinically, metformin was demonstrated to be helpful in improving OS in stage II-III CRC in adjuvant settings [43, 44]. In addition, for CRHT, a recent study indicated that administration of pegfilgrastim on the same day as capecitabine-based chemotherapy in gastrointestinal cancers was safe, as it did not require dose reductions or cause a chemotherapy delay [45]. However, it is also noteworthy that certain causes, such as CIA, are still difficult to manage, irrespective that red blood cell transfusion, erythropoietin-stimulating agents (ESAs) and intravenous iron have been extensively studied in the clinic [46]. Although these approaches were found to play an important role in improving the quality of life of patients who underwent chemotherapy, approaches including blood transfusion or intravenous iron were not helpful in improving the outcome [47, 48]. For ESAs, it was found that approximately 1/3 of the patients did not respond to the treatment alone [49]; moreover, responses were not observed until 4–6 w after therapy initiation even in the responding ones [50]. Based on these results, a tailored therapeutic strategy is of pivotal importance for patients presenting with IDacT caused by CIA. Interestingly, in previous studies, a 12-m duration of single capecitabine ACT was found to yield better DFS and OS results than a 6-m duration in selected patients [51]; and a reduced dosage of oxaliplatin in the FOLFOX regimen for ACT had no obvious impact on DFS and OS [52]. Thus, we speculate that a duration extension with a dosage reduction may be acceptable for these patients. Nonetheless, these speculations need to be validated in prospective studies in the future.

There are some limitations to our study. First, it was a retrospective study that was performed in a single medical center, and the small sample size could lead to biased findings. Second, stage II cases (*n* = 14) without any of the risk factors were also included in our study; although previous studies have shown that these patients would also benefit from ACT [53–55], a standardized ACT and duration were not well established for such cases [56]. Third, 20 patients with at least one tumor deposit were included (II: *n* = 2, III: *n* = 18). Some studies indicated that stage I-III cases with these features would behave similarly to stage IV patients and would have no DFS benefits from ACT, particularly in stage III cases [57, 58]. Based on these studies, patients with these features would exhibit no differences in DFS regardless of the IDacT in our cohort. However, larger sample studies with restricted inclusion criteria would overcome these limitations in the future.

## Conclusion

Overall, our study indicated that IDacT impaired survival in stage II-III CRC patients. Although TD and DpC were significant in predicting survival, DpC was an independent prognostic factor, and patients who experienced DpC for a long period had significantly worse DFS.

## Data Availability

The datasets generated during the current study are not publicly available due to limitations of ethical approval involving the patient data and anonymity but are available from the corresponding author on reasonable request.

## Abbreviations

ACT: Adjuvant chemotherapy
CRC: Colorectal cancer
IDacT: Irregular delay of adjuvant chemotherapy
TD: Total delay
DpC: Delay *per* cycle
DFS: Disease free survival
ROC: Receiver operating characteristic curve
XELOX: Oxalipaltin + capecitabine
FOLFOX: Oxalipaltin + leucovorin + 5-fluorouracil
DFS: Disease free survival
OS: Overall survival
AEs: Adverse effects
K-M: Kaplan–Meier
5-Fu: 5-Fluorouracil
CRHT: Chemotherapy-related hematological toxicities
CIA: Chemotherapy-induced anemia
ESAs: Erythropoietin-stimulating agents
